# Developmental Expression of Claudins in the Mammary Gland

**DOI:** 10.1007/s10911-017-9379-6

**Published:** 2017-04-28

**Authors:** Heidi K. Baumgartner, Michael C. Rudolph, Palaniappian Ramanathan, Valerie Burns, Patricia Webb, Benjamin G. Bitler, Torsten Stein, Ken Kobayashi, Margaret C. Neville

**Affiliations:** 1 Department of Obstetrics and Gynecology, University of Colorado Denver, Aurora, CO 80045 USA; 2 Division of Endocrinology, Metabolism & Diabetes, University of Colorado Denver, Aurora, CO 80045 USA; 3 Department of Pathology, University of Texas Medical Branch at Galveston, Galveston, TX 77555 USA; 4 Department of Physiology and Biophysics, Anschutz Medical Center, University of Colorado Denver, Aurora, CO 80045 USA; 5 College of Medical, Veterinary and Life Sciences, University of Glasgow, Glasgow, UK; 6 Research Faculty of Agriculture, Hokkaido University, Sapporo, 060-8589 Japan; 7 6561 Glencoe St., Centennial, CO 80121 USA

**Keywords:** Mammary gland, Claudin-1, Claudin-3, Claudin-4, Claudin-7, Claudin-8, Extra-junctional claudin, Involution, Infection

## Abstract

Claudins are a large family of membrane proteins whose classic function is to regulate the permeability of tight junctions in epithelia. They are tetraspanins, with four alpha-helices crossing the membrane, two extracellular loops, a short cytoplasmic N-terminus and a longer and more variable C-terminus. The extracellular ends of the helices are known to undergo side-to-side (*cis*) interactions that allow the formation of claudin polymers in the plane of the membrane. The extracellular loops also engage in head-to-head (*trans*) interactions thought to mediate the formation of tight junctions. However, claudins are also present in intracellular structures, thought to be vesicles, with less well-characterized functions. Here, we briefly review our current understanding of claudin structure and function followed by an examination of changes in claudin mRNA and protein expression and localization through mammary gland development. Claudins-1, 3, 4, 7, and 8 are the five most prominent members of the claudin family in the mouse mammary gland, with varied abundance and intracellular localization during the different stages of post-pubertal development. Claudin-1 is clearly localized to tight junctions in mammary ducts in non-pregnant non-lactating animals. Cytoplasmic puncta that stain for claudin-7 are present throughout development. During pregnancy claudin-3 is localized both to the tight junction and basolaterally while claudin-4 is found only in sparse puncta. In the lactating mouse both claudin-3 and claudin-8 are localized at the tight junction where they may be important in forming the paracellular barrier. At involution and under challenge by lipopolysaccharide claudins −1, −3, and −4 are significantly upregulated. Claudin-3 is still colocalized with tight junction molecules but is also distributed through the cytoplasm as is claudin-4. These largely descriptive data provide the essential framework for future mechanistic studies of the function and regulation of mammary epithelial cell claudins.

**Electronic supplementary material:**

The online version of this article (doi:10.1007/s10911-017-9379-6) contains supplementary material, which is available to authorized users.

## Part 1. An Overview of Claudin Structure and Biology

### Introduction

**Fig. 1:**
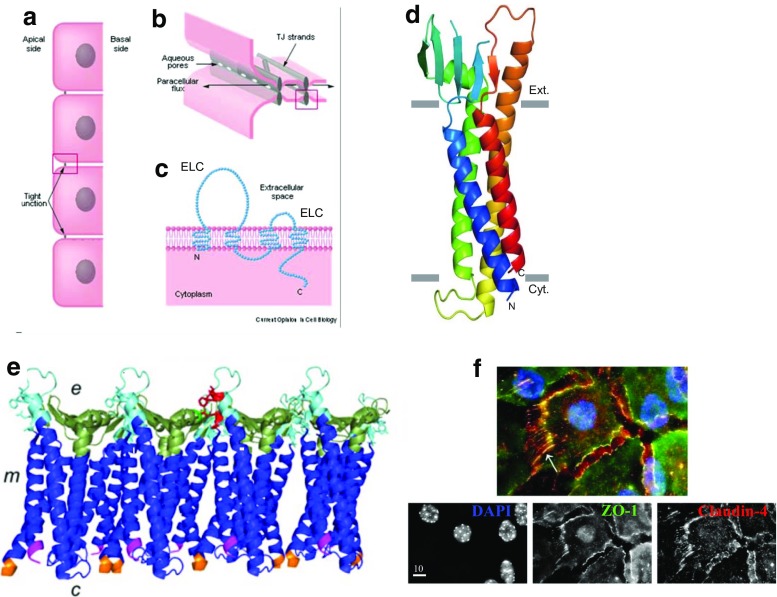
Localization and molecular structure of claudins. **a** Model mucosal epithelial layer showing apical location of tight junctions. **b** Aqueous pores formed by some claudins in the tight junction provide a pathway for paracellular ionic flux. **c**. Diagrammatic view of a claudin molecule in the plasma membrane showing the four transepithelial regions, the two extracellular loops (ECS) and the cytoplasmic N and C termini. **a**,**b**,**c**, from [23], *Current Opinion in Cell Biology*. **d** Detailed crystal structure of the claudin-15 molecule showing its orientation in the plasma membrane, represented by grey rectangles; image from ref. [76], *Science*; used by permission. Transmembrane segments (TM) are shown as alpha-helices with TM1 in *blue*, TM2 in *green* with the four beta-sheets of extracellular loop 1 between them. TM3 is *yellow* with the helix extending beyond the membrane into the interstitial space; TM4 is *red* and ECL2 is between TM3 and TM4 and contains one beta-sheet. The C-terminus of the molecule was removed to facilitate crystallization and is not shown. **e** Claudins are thought to polymerize in the membrane via interactions between amino acids on membrane adjacent portions of ECL1 (*bright green*) and ECL2 (*red*). Image shows claudin-15 polymer with extracellular side (*e*), cytoplasmic side (*c*), and membrane (*m*) Image from [10], *The Biochenical Journal*. **f** Immunofluorescent localization of claudin-4 in subconfluent cultured primary mammary epithelial cells. Upper image, colocalization of claudin-4 (*red*) with the tight junction protein ZO-1 (*green*) and nuclei (*blue*). Claudin-4 is colocalized with ZO-1 in the tight junctions; it is also present as cytoplasmic puncta and in cellular projections that show no sign of ZO-1 staining. Image from ref. [18], *BMC Cell Biology*

The claudin family comprises a large class of transmembrane proteins whose activity was first described in 1998 by Tsukita and colleagues who showed that claudin-1 and claudin-2 are important participants in tight junctions [1], interacting between cells to form the apical barrier that controls epithelial permeability (Fig. 1a). At least 63 different claudin family members have been identified since their initial discovery [2]; about 27 of these molecules are thought to be present in humans [3]. All are tetraspanins with four transmembrane domains, intracellular N and C termini, and two extracellular loops (Fig. 1c). Early studies from the Tsukita laboratory showed that expression of claudin-1 or claudin-2 in cultured fibroblasts leads to formation of continuous interacting polymeric rows of claudin similar to the polymeric claudins observed in epithelial tight junctions [4], providing positive proof of the importance of claudins in these structures. Genomic knockdown of claudin-1 in mice led to death at birth due to defects in the epidermis establishing that claudins are important in both mucosal and epidermal barriers [5].

Claudins have been most studied as components of the epithelial tight junction where they can inhibit transcellular permeation of small molecules or form specific channels allow flux of anions or cations across the paracellular space of the epithelium (Fig. 1b). In particular, claudins-2, −10b, and −15 have been shown to mediate cation permeation, while claudins-10a and -17 mediate anion permeation [6, 7]. Claudin-2 also allows water to permeate the epithelial barrier [6]. The crystal structure of pore forming claudin-15 has recently been published [8] showing that the four transmembrane (TM) segments of the molecules form alpha-helices while extracellular loop 1 (ECL1), involved in intercellular interactions, possesses four beta-pleated sheets and the smaller extracellular loop 2 (ECL2) has one (Fig. 1c, d). Studies of claudin-4 bound to the *Clostridium perfringens* endotoxin (CPE) are consistent with this structure [9]. Elegant studies of interacting molecules using FRET technology have shown that claudins possess both side-to-side (*cis*) interactions with other claudins in the same membrane and head-to-head (*trans)* interactions with claudins in adjacent cells [10].

X-ray crystallographic studies of the binding of *Clostridium perfringens* endotoxin (CPE) with claudin-19 [9, 11] shed light on the *cis* interactions: in this claudin the leucine on TM segment 1 interacts hydrophobically with phenylalanines on TM1 and TM3 on an adjacent molecule (Fig. 1e). Five amino acid peptide mimics to the extracellular helix of TM3 in claudins-3 and -4 disrupted epithelial permeability and claudin-4 localization in cultured Eph4 mammary epithelial cells (peptide sequence DFYNP, amino acids 146–150 on TM3) [12]. Recent work from the Kovak laboratory showed that lung alveolar epithelial cells isolated from alcohol treated rats contain claudin-5, a molecule usually found in the endothelium [13]. Treatment of these cells with a peptide mimic to the same TM3 locus on claudin-5 (peptide sequence EFYDP) decreased trans-epithelial resistance and increased the permeability of large molecules when these cells were grown on Transwell permeable filters [14]. These independent findings using two peptides targeting two different claudins indicate that *cis* interactions of the claudin molecule are important in maintaining tight junction structure.

Claudins also engage in interactions with the cytoskeleton mostly through the YV-amino acid motif at the C-terminus of most claudins. This motif allows interactions with PDZ domain containing proteins such as ZO-1-2, and −3, components of the tight junction [15]. These molecules in turn bind to the actin cytoskeleton and to regulatory molecules which control not only tight junction formation but also cell polarity, migration, proliferation and apoptosis [16]. As an example, we have shown that treatment of cultured cells with the DFYNP peptide, mentioned above, decreases cell motility and increases apoptosis through the FAS-dependent extrinsic pathway [12, 17, 18]. Another tetraspanin, occludin, is exclusively localized to the tight junction in normal epithelia, but its function is not entirely clear as occludin knockout mice survive with intact epithelial structures [19]. Altogether the claudin family of tetraspanin proteins provides important epithelial barrier functions; emerging evidence reviewed below indicates they may also participate in additional intracellular and extracellular interactions.

### Tissue Expression Patterns of Claudins

Figure 1f showing claudin-4 localization in subconfluent cultured primary mammary epithelial cells illustrates the complexity of claudin localization. In cultured cells the molecule does colocalize with ZO-1 in tight junctions. However, it is also clearly seen in small cytoplasmic puncta and in projections from the cell reaching out both to the extracellular matrix and to an adjacent cell. This image raises several important questions about claudin function to which we do not have satisfactory answers. We briefly summarize claudin expression and localization in several epithelial tissues in this introduction providing a cell biological background for more detailed analysis of our current understanding of claudin expression and localization in the mammary gland.

Epithelial claudin composition changes both with differentiation and position in mucosal organs, a process termed “claudin switching” by Capaldo and Nusrat [20]. In the kidney claudin localization was determined by co-immunostaining with segment-specific molecules such as aquaporins-1 and -2; a very complex distribution was observed depending on the segment of the renal tubule [21, 22]. For example, while claudin-2 was found in most segments, claudin-16, which has been associated with diseases of magnesium transport [23], was present only in the thick ascending limb of the loop of Henle. The distribution of claudin-10 depended on isoform: isoform 10a formed a paracellular channel permeable to anions whereas isoform 10b formed a cation-selective channel [7]. In the digestive system claudin-3 was localized to the tight junctions of the intestinal epithelium and the liver, while claudin-4 was found at junctions in the epithelium of the stomach and colon [24]. In addition some claudins have been shown to be localized in cytoplasmic “dots” or puncta, presumably vesicles, distributed in the cytoplasm [3]. For example, claudin-3 appeared to be extra-junctional in the stomach and duodenum as well as the pancreas [24] and claudin-4 was mainly localized apically in small intestinal cells preparing to slough into the lumen and demonstrating little overlap with the tight junction marker ZO-1. In the epididymis claudin-1 was found in the lateral and basal borders of the principal cells; electron microscopic images showed it to be localized at tight junctions, along lateral borders and, unexpectedly, associated with the basement membrane [25].

While the tight junction claudin pool has received the most attention, many cells have two or more pools of claudins; the tight junction pool and a cytoplasmic pool often observed as puncta, presumably vesicles, distributed at the basolateral borders of the cell [26]. The first clear demonstration of cytoplasmic claudin-3 was in Eph4 and MDCK cells transfected with GFP-NClaudin-3, where both native claudin-3 and GFP-Nclaudin-3 were found both in tight junctions and in what the authors designated as cytoplasmic granules, postulated to be vesicular structures [27]. Some of these granules appeared to originate from endocytosis of tight junction claudin-3; in some cases the granules were associated with late lysosomal markers. In studies of *Clostridium perfringens* endotoxin binding to claudin-3 in HEK cells, CPE was found to bind to claudin-3 on cell surfaces, not associated with the tight junction [28]. This finding suggests that non-junctional claudin in epithelial cells cycles to the cell surface independent of tight junction interactions and may not simply reflect transport to and from the tight junctions. More recent work has shed some light on the interactions of non-junctional claudins-4 and -7 with intracellular and membrane proteins as we will elaborate below.

As will be shown in detail in later sections of this paper the classical claudins-1, -3, -4, -7, -8, all non-pore-forming claudins, are present at various times and cellular positions in the mammary epithelium showing marked developmental switching. Claudins-1, -3 and -8 are clearly localized to tight junctions at various stages of mammary development. However, claudins-3, -4 and -7 are also present at the basal and lateral surfaces of the ductal epithelial cells where they can be seen as cytoplasmic puncta not colocalized with the tight junction protein ZO-1. We briefly review work from other tissues that sheds some light on the interactions and functions of these non-junctional claudins. We start with claudin-7 where the most definitive work is available, then continue to claudins-3 and -4.

### Claudin-7

Although transduced claudin-7 has been shown to form polymerized rows in S7 cells, a fibroblast line lacking tight junctions, in vivo the molecule was generally not associated with tight junctions but was found in cytoplasmic puncta, which are likely to be vesicles [29, 30]. We and Kobayashi showed previously that claudin-7 is present in alveolar cells at all stages of mammary development [29, 31] localized to basolateral surfaces as it is in many other tissues like the epididymis and intestine [30, 32, 33]. Recently, claudin-7 was shown to complex with EpCAM, an epithelial adhesion molecule enriched at the basolateral membrane of intestinal and other cells [34]. When EpCAM was knocked down in intestinal cells, the small amount of claudin -7 remaining was localized to tight junctions. More recent work from the Thuma laboratory [35, 36] clearly showed that palmitoylated claudin-7 recruits EpCAM to glycolipid-enriched membrane domains, often designated as lipid rafts. Only when a mutation that prevents palmitoylation of claudin-7 was present, did the protein associate with tight junctions. Studies of intestinal architecture in claudin-7 knockout mice led Ding and associates [33] to conclude that claudin-7, interacting with EpCAM and integrin-α2 mediates lateral cell-cell interactions as well as interactions with the cell-matrix.

### Non-junctional Claudins-3 and -4

Workers in the Tsukita lab found more than a decade ago that claudins-3 and -4 could be localized in cytoplasmic vesicles whose presence was apparently related to cell motility [27]. In most cells and tissues claudin-3 has been almost exclusively described as being part of the tight junction; however, it is notable that during maturation of the murine intestine during the first 3 weeks of life basolateral claudin-3 increases simultaneously with its localization at the apical membrane of the intestinal cells [37]. In the colonic epithelium of mature C57Bl/6j mice claudin-3 is localized entirely at the basolateral membrane [38]. Because claudin-3 has been seen to move in and out of the tight junctions [20], cytoplasmic vesicles have often been considered as storage depots for tight junction claudins.

Similarly, in MDCK cells claudin-4 is found both at tight junctions and in discrete cytoplasmic structures. Biotin ligase fused to the N-terminus of claudin-4 or to the tight junction protein occludin, was used to biotinylate proteins proximal to both occludin and claudin-4 followed by purification of biotinylated proteins and identification by mass spectrometry [39]. Using the data from this study of MDCK cells we have divided the biotinylated proteins into two categories: Category 1 contains proteins that were biotinylated when biotin ligase was fused to either occludin or claudin-4, presumably identifying proteins associated with the tight junction. Category 2 contains proteins only biotinylated when biotin ligase was fused to claudin-4. Interestingly, category 1 includes vesicle proteins involved in fusion with the plasma membrane in the neuronal synapse: synaptobrevin like protein YKT6-like, vesicle associated membrane protein VAMP-2, vesicle associate membrane protein VAMP-5, synaptosomal associated protein SNAP-23, a regulatory factor and syntaxins-6 and-8. CAR (coxsackievirus and adenovirus receptor homolog) is also included in this category. These observations suggest that synaptic-like vesicles may be involved in claudin cycling to the tight junction as proposed by Nusrat and colleagues in a symposium article reporting that SNARE proteins may be associated with recycling of tight junction molecules [40]. In addition recent comprehensive experiments showed that syntaxin-8 binds to claudin-16 and promotes the exocytosis of claudin-16 from subcellular compartments to the tight junctions [41]. The distribution of SNARE proteins in the lactating mammary epithelium has recently been summarized [42], how this distribution relates to claudin distribution is a subject for future research.

Category 2 proteins include Rab7a as well as cell adhesion molecules, CD44 antigen, CEA1, integrins β and α2 and cytoskeletal related proteins BAI1 and Marcks-related protein. These findings lead to a second hypothesis that claudin-4 localized to cytoplasmic vesicles is involved in cell-cell and cell-matrix interactions, potentially placing claudins as integrators of both cell-extrinsic and cell-intrinsic signal transduction pathways. This hypothesis is supported by the finding in cultures of normal and T47D breast cancer cells cultured on collagen that knockdown of claudin-4 or treatment with the claudin disrupting peptide DFYNP decreased cell motility and increased apoptosis [18]. To further test this hypothesis localization of claudins during the various stages of mammary development as well as identification of interacting proteins will be necessary. In the next section we provide a comprehensive review of claudin expression and localization during mammary development; work on interacting proteins remains for the future.

## Part 2: Claudin Expression and Localization during Mammary Gland Development

Because the role of claudins in mammary development has been extensively studied in the mouse, and considerable published data is available from both the Kobayashi [31, 43] and the Neville [12, 29, 44–47] laboratories on tight junction function and claudins, we focus on murine data in this paper. Here we combine published data from these and other laboratories with unpublished data from the Neville laboratory offering a descriptive framework that should foster future mechanistic studies on claudin regulation and function throughout mammary development.

### Claudin mRNA Expression during Development in the Murine Mammary Gland

**Fig. 2:**
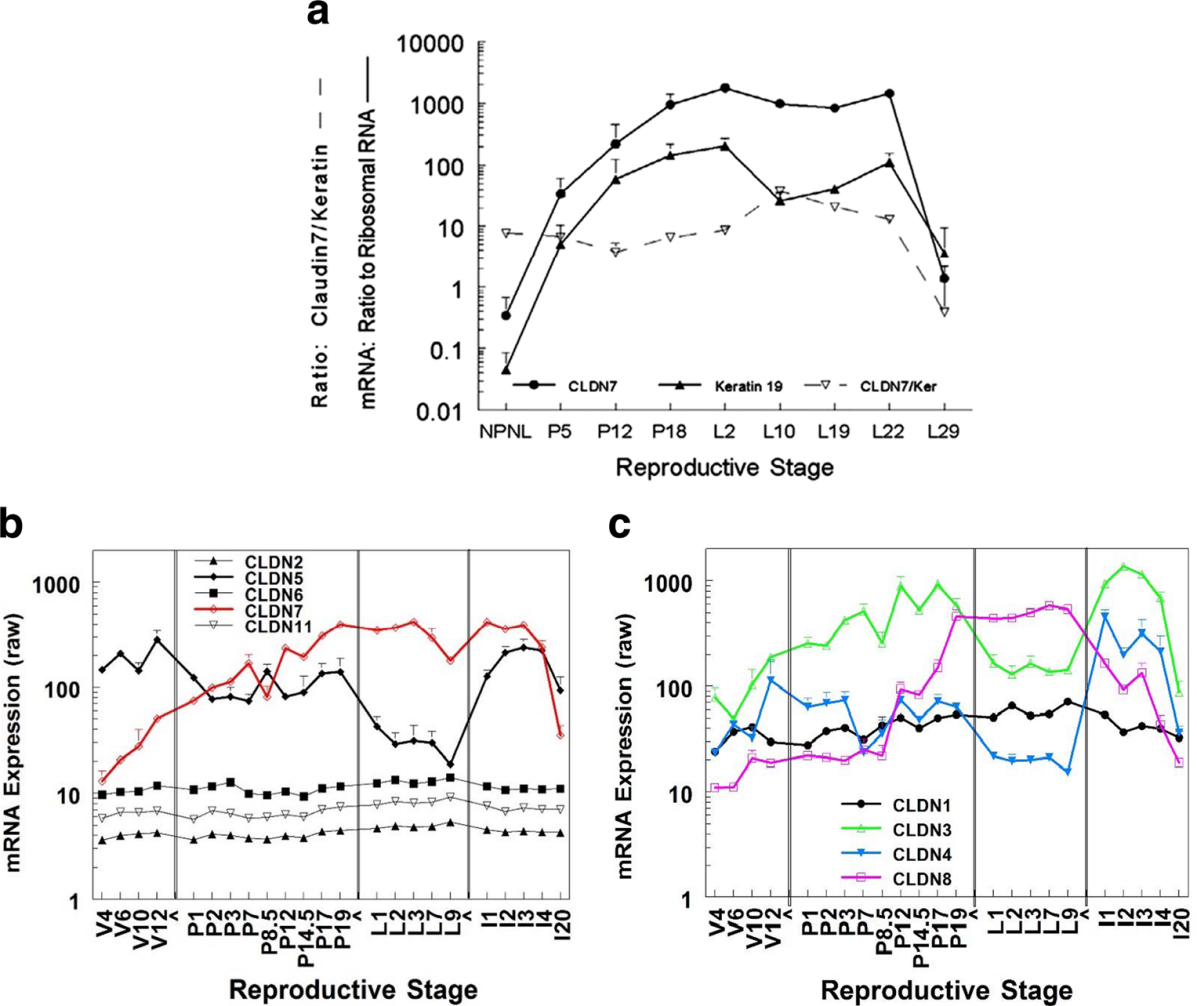
Developmental expression of claudin mRNA in the murine mammary gland. **a** Developmental expression of claudin-7 compared to expression of the epithelial keratin 19 in glands of CD1 mice using real time PCR. Graph from Reference [29] *Breast Cancer Research.* **b, c**. Time course data obtained from Affymetrix arrays (MG-U74Av2--able to hybridize 12,000 genes) performed in the Gusterson [48] and Neville [49] laboratories. Data are combined to demonstrate how these claudins vary through the reproductive cycle. V, glands isolated from non-pregnant non-lactating mice at 4, 6, 10 and 12 weeks after birth. P (pregnancy), glands isolated 0, 1, 2, 3, 7, 10.5, 12, 14.5, 17 and 19 days after mating. Birth occurs about 19.5 days after mating in these two strains. L (lactating), glands isolated at 1, 2, 3, 7 and 9 days after parturition. I, (involution) glands 1, 2, 3, 4 and 20 days after pup removal on day 8 ([48]) or 10 ([49]) of lactation. **b**. Expression of claudins-2, 5, 6, and 11 compared to that of claudin-7. **c**.Expression of claudins-1, −3, −4, and −8 as indicated

Published work from the Neville laboratory [29] showed the expression of claudin-7 mRNA through development (Fig. 2a). The expression of both claudin-7 and keratin 19, a marker of alveolar epithelial cells in the mammary gland, was analyzed by quantitative PCR (qPCR). The parallel expression of the two genes suggests that claudin-7 expression can be used as a marker of the proportion of the tissue occupied by epithelial cells. These data indicate that the proportion of epithelial tissue increases about 1000-fold between the virgin and lactating states, an important consideration when whole mammary tissue is being analyzed for mRNA or protein.

Two previously published microarray studies using RNA extracts from whole tissue [48, 49] provide an overall picture of epithelial claudin mRNA expression as the murine mammary gland passes from the virgin state, through pregnancy, lactation and involution (Fig. 2b, c). Figure 2b shows three claudins (-2, -6, -11) expressed at low and invariant levels through development compared to the expression of claudins-5 and -7. There is no evidence that claudin-2, 6 and -11 proteins are found in the mammary gland. Claudin-5 is confined to the endothelium in most tissues [13]; we have not been able to observe it within the mammary epithelium (unpublished data). Claudin-7 expression was consistent with our previous findings [29]. Claudin-1 (Fig. 2c) mRNA was expressed at a relatively constant level throughout the reproductive cycle. Claudin-3 mRNA expression roughly paralleled that of claudin-7 in glands from virgin and pregnant animals. Its expression fell about 8-fold at the onset of lactation possibly reflecting the loss of non-junctional claudin-3; it still remained at a relatively high level through lactation as expected from its localization at tight junctions (see below). It rebounded with involution. Claudin-4 was expressed at modest levels in the glands from virgin and pregnant animals, falling to almost undetectable levels during lactation; it increased more than 10-fold after pup removal. Claudin-8 has been little studied but levels of its mRNA increased about 30-fold between the virgin and lactating state; it remained high through lactation and decreased with cessation of milk secretion. Levels of claudins-1, -3, -4 and -7 mRNA, as well as their protein levels, measured by the Plante laboratory over murine mammary development [50], are consistent with the values shown in Fig. 2.

### Claudin Localization in Mammary Glands of Non-Pregnant, Non-Lactating Animals

**Fig. 3:**
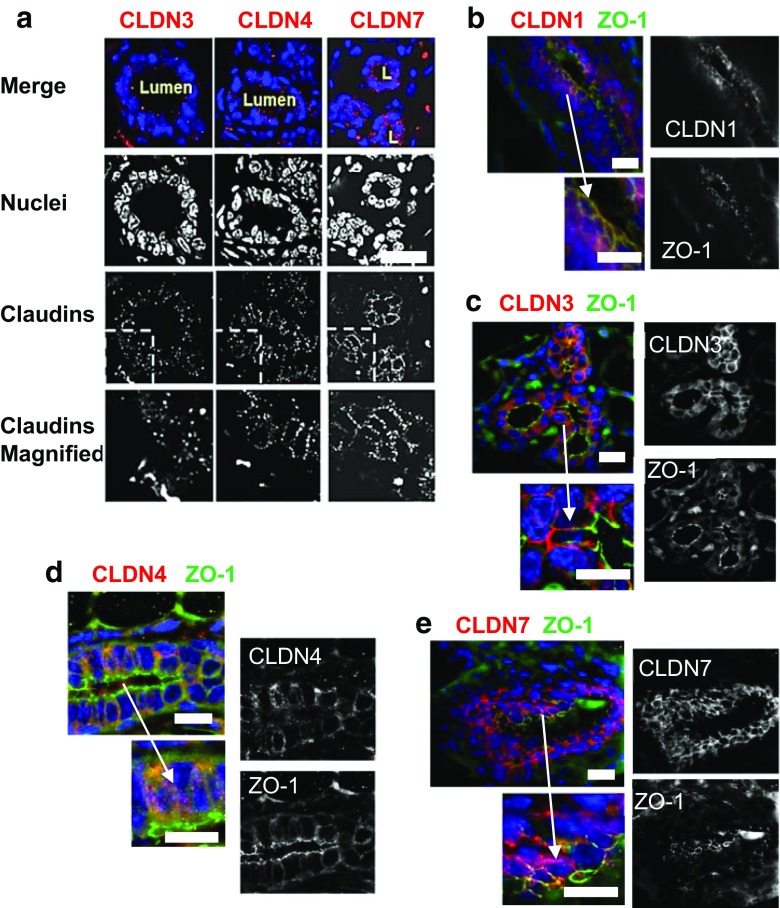
Immunofluorescence analysis of claudins in the mammary gland of the non-pregnant, non-lactating mouse. **a.** Immunofluorescence analysis of Claudins-3, −4, and −7 in mammary glands of the FVB mouse highlighting the punctate nature of the stain localized in the ductal cells. **b, c, d.** Immunofluorescent images of ductal sections mammary glands from CD1 virgin mice stained for claudins-1, −3, −4 respectively (*red stain*) and the tight junction protein ZO-1 (*green*). **b**. Claudin-1 overlapped heavily with ZO-1, appearing yellow in merged images, indicating association with tight junctions in this ductal section. **c**. Claudin-3 was heavily stained along lateral and basal borders but showed little overlap with ZO-1. **d**. Claudin-4 was observed as sparse cytoplasmic puncta, again with little overlap with ZO-1. **e**. Claudin-7 was localized along the basal and lateral border of the ductal cells in the CD1 mouse with little or no overlap with ZO-1 See also ref. [29]. Scale bars, 20 μm. See Methods section for tissue preparation and staining methods for all these figures

Mammary development begins at puberty with estrogen-stimulated ductal lengthening and branching [51]. Claudin-1 was located at the tight junctions of the mammary ducts [29, 52–54] (Fig. 3b), presumably restricting paracellular permeability although no permeability data appear to be available. Claudin-1 has been clearly observed in mammary epithelia from 35 day old rats as well [54].

Claudins-3, -4 and -7 were observed as cytoplasmic puncta at the basal and lateral surfaces of ductal epithelial cells in sections from mammary glands of non-pregnant non-lactating mice (Fig. 3a). The most intense claudin-3 stain was seen in regions near the basolateral membrane showing minimal overlap with the tight junction protein ZO-1 (Fig. 3c). Distinct claudin-3 puncta could also be seen in the perinuclear regions. Claudin-4 was present only in lightly-stained sparse intracellular puncta (Fig. 3d). Again overlap with ZO-1 was minimal. As shown previously [29], claudin-7 was not associated with ZO-1 (Fig. 3e). Together, these observations indicate that extra-junctional patterning of claudin-3, -4, and -7 is common in the mammary epithelium and suggest that claudins-3, -4 and -7 are likely to have a function other than sealing of the tight junction.

Claudins-1, -3, -4 and 7 are the four most studied claudins in normal mammary glands of humans [52] and other animals. Kominsky et al. [55] saw little claudin-3 and claudin-4 in tissues adjacent to breast tumors in the human mammary gland but Tokes et al. [52] found that these claudins were often present in the normal breast. In normal human breast tissue adjacent to breast cancer lesions claudins-1 and -3 were clearly localized apically with some basal staining by immunofluorescence [56]**.** In canine mammary glands the mRNA for CLDN-1 and -7 were the most highly expressed with claudins-3 and -4 moderately lower [53]. Cellular localization of these canine claudins was unexpectedly found to be along the entire lateral area of the epithelial cells and not just at the apical border. Plante and colleagues observed basolateral staining of claudins-3 and -7 in mammary glands from virgin mice with apical localization only at pregnancy day 18 [50].

### Claudin Expression and Localization in Mammary Glands from Pregnant Mice

**Fig. 4:**
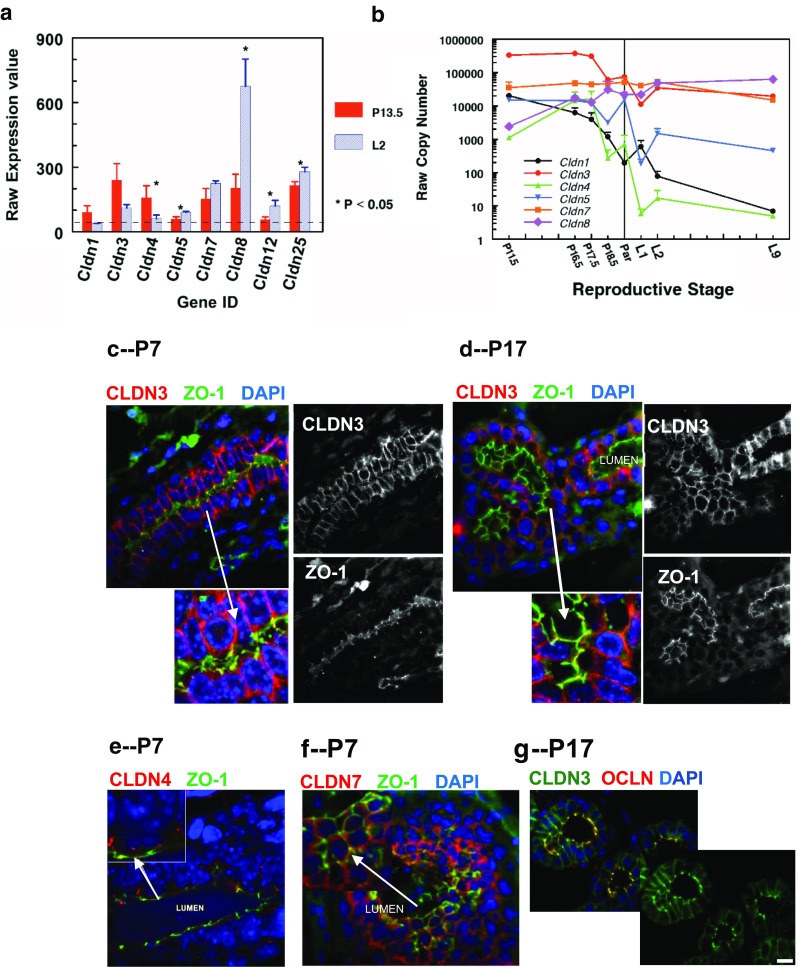
Expression and localization of claudins in pregnancy. **a**. Microarray analysis of claudin mRNA in MECs isolated from pregnant (day 13.5) and lactating (day 2) FVB mice [58]. MECs were isolated from the 4th mammary gland according to the techniques of Rudolph and colleagues [58]. mRNA expression was analyzed using Affymetrix MoGene_1_0-st-v1 chip arrays [77]. Raw expression values for all claudins with expression values above 40 (*dotted line*) at pregnancy day 13.5 (*red*) or lactation day 2 (*blue*) are shown. * Expression significantly different between pregnancy and lactation, *P* < 0.05 **b**. mRNA expression of claudins 1, 3, 4, 5, 7 and 8 during the transition from pregnancy to lactation by quantitative real time PCR using lysates of whole mammary glands (See Methods, Fig. 4). **d,e.** Immunofluorescence localization of claudins-3, −4 and −7 in mammary glands from CD1 pregnant mice (Neville laboratory, see Methods section). **c,e,f**. Pregnancy day 7. Similar results were observed in both formalin-fixed (shown) and frozen (not shown) sections. **d,g**. Pregnancy day 17. **g**. Localization of claudin-3 in the mammary gland from the ICR mouse; in this strain claudin-3 was localized both basolaterally and with occludin at pregnancy day 17 [43]. **c-g**. Scale bars, 20 μm

Increases in progesterone as pregnancy commences stimulate alveolar proliferation followed by differentiation, a process also stimulated by placental lactogen (and prolactin in humans) [57]. To validate the expression of mammary claudins, mammary alveolar epithelial cells (MEC) were isolated [58] from the mammary glands of pregnant day 13.5 and lactation day 2 FVB mice and gene expression evaluated by advanced microarray analyzing nearly 30,000 genes (Fig. 4a). As previously observed (Fig. 2) the mRNA for claudins-1, -3, -4, -5, -7, and -8 were found to be present at significant levels in pregnancy or lactation or both. The non-canonical claudins-12 and -25 were also expressed at levels above background in both pregnancy and lactation with significant increases during lactation. There is a little data on mammary claudin-12; it showed weak cytoplasmic staining in normal breast cells adjacent to breast tumors, but was upregulated in some tumors [59]. Its expression increased MCF-7 cell migration [60], but nothing is currently known about its function in the normal mammary epithelium. This is the first report of claudin-25 in the mammary gland and nothing more is known at this writing.

Secretory activation is set in motion by a decrease in progesterone associated with parturition and has been shown to require both prolactin and glucocorticoid signaling [61]. Figure 4b shows detailed changes in expression of classical claudins around parturition in the mouse; the use of qPCR allows comparison of expression levels. Close examination shows that the transition to lactation levels for most of these genes starts at pregnancy day 18.5, just when progesterone falls. At mid-pregnancy claudin-3 mRNA was present at the highest levels. The protein showed heavy basolateral staining in both early and late pregnancy (Fig. 3c–e). By pregnancy day 17 it was also localized with ZO-1 (CD1 mice, Fig. 4d) or occludin (ICR mice, Fig. 4g, ref. [43]). Claudin-4 mRNA was much lower and fell almost 100 fold on P18.5. The protein was present as sparse cytoplasmic puncta at P7. The mRNA for claudin-5, the endothelial protein, fell in early lactation probably reflecting the decrease in the amount of adipose tissue, which is heavily vascularized. Claudin-7 mRNA remained approximately constant and continued to be localized basolaterally (Fig. 4f). Claudin-8 was fairly low at mid-pregnancy but rose 10-fold by P18.5 and another 3-fold in early lactation to become the most highly expressed claudin gene. We show in the next section that it is localized at the tight junction suggesting that it may help establish the paracellular barrier present in the lactating gland [62].

### Claudins in the Lactating Mammary Gland

The analyses of claudin mRNA in the mammary glands from lactating mice (Fig. 2, Fig. 4a, b) suggested that the most important epithelial claudins at this time are claudins-3, -7, -8. The mRNA for claudin-3 fell about 10-fold at the onset of lactation, but was still present at significant levels. The mRNA for claudin-7 was maintained at high levels throughout. The mRNA for claudin-8 increased more than 25-fold to become the most highly expressed claudin mRNA in the lactating gland (Fig. 4b).

**Fig. 5:**
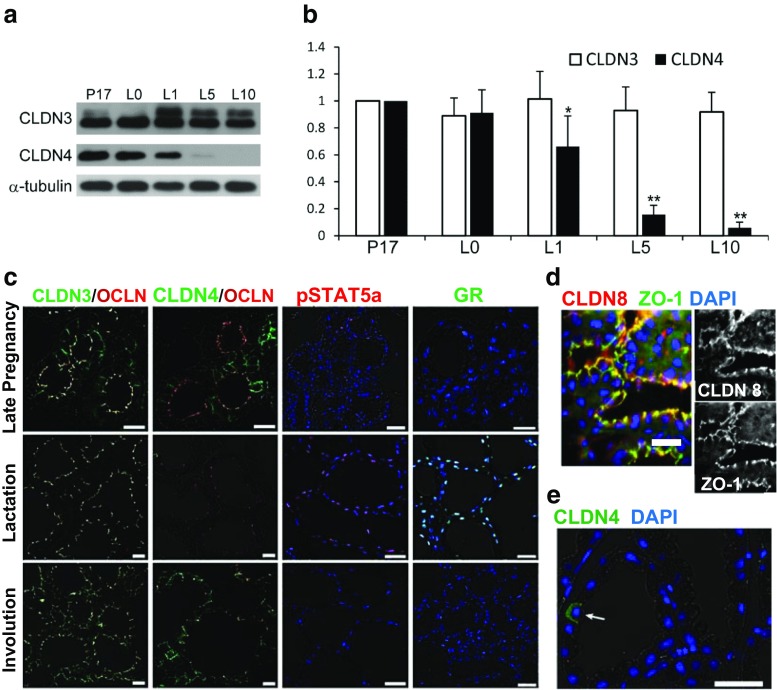
Protein expression and localization for claudins in the mammary gland of mice in mid-lactation. **a** Western blots for claudins-3 and -4 in the lactating gland at days 0, 1, 5, and 10 compared to late pregnancy (P17). Alpha-tubulin was the loading control. **b** Quantitation of Western blots for claudin-3 and claudin-4 compared to late pregnancy (P17). Significantly different from P17, *P < 0.05; **P < 0.01. **c**. Subcellular localization of claudins-3 and -4, pSTAT5a and the glucocorticoid receptor (GR) in the murine mammary gland at day 10 of lactation. Paraffin sections were immunostained with antibodies to the relevant claudin (*green*), occludin (*red*), pSTAT5a (red) and GR (*green*). *Blue*; nuclei stained with DAPI. **d** Immunofluorescence analysis of claudin-8 from the mammary gland of a day 2 lactating mouse. **e**. Rare occurrence of basolateral CLDN4 in a mammary alveolar cell of a lactating mouse. *Scale bars*; 20 μm. Images **a** and **b** based on ref. [43], image **c** based on Ref [64] and image **e** from Ref [31] *Plos One*, all ICR mice. Image **d**, Neville laboratory (CD1 mouse; see methods)

Western blots from the Kobayashi laboratory [43] showed that claudin-3 protein in the late pregnant and lactating gland is relatively constant, whereas claudin-4 protein decreased rapidly after the onset of lactation (Fig. 5a, b). The presence of a higher molecular weight form of claudin-3 during lactation suggests that phosphorylation, possibly on threonine 192 [63], or palmitoylation [36], may be important for tight junction sealing. The nature of this higher molecular weight form requires more investigation. Immunofluorescence analyses showed claudin-3 localized to tight junctions during lactation (Fig. 5c) [31, 64]. The basolateral claudin-3, present in pregnancy, was no longer apparent. Claudin-4 was largely absent from the lactating epithelium (Fig. 5c) except for very occasional cells showing strong basolateral staining (Fig. 5e). As at other stages claudin-7 was found in puncta near the basal and lateral borders of the alveolar epithelial cells from both the Neville [29] and Kobayashi [31] laboratories (not shown). Claudin-8 protein, shown to reduce paracellular permeability in kidney cells [65], was localized with ZO-1 in the lactating gland (Fig. 5d). Together these findings lead to the hypothesis that junctional claudins-3 and -8 are responsible for the very low paracellular permeability of the lactating mammary gland [66]. Both the regulatory molecules pSTAT5a and the glucocorticoid receptor (GR), thought to be important controllers of milk secretion [67, 68], were associated with nuclei only in sections from the lactating gland (Fig. 5c).

**Fig. 6:**
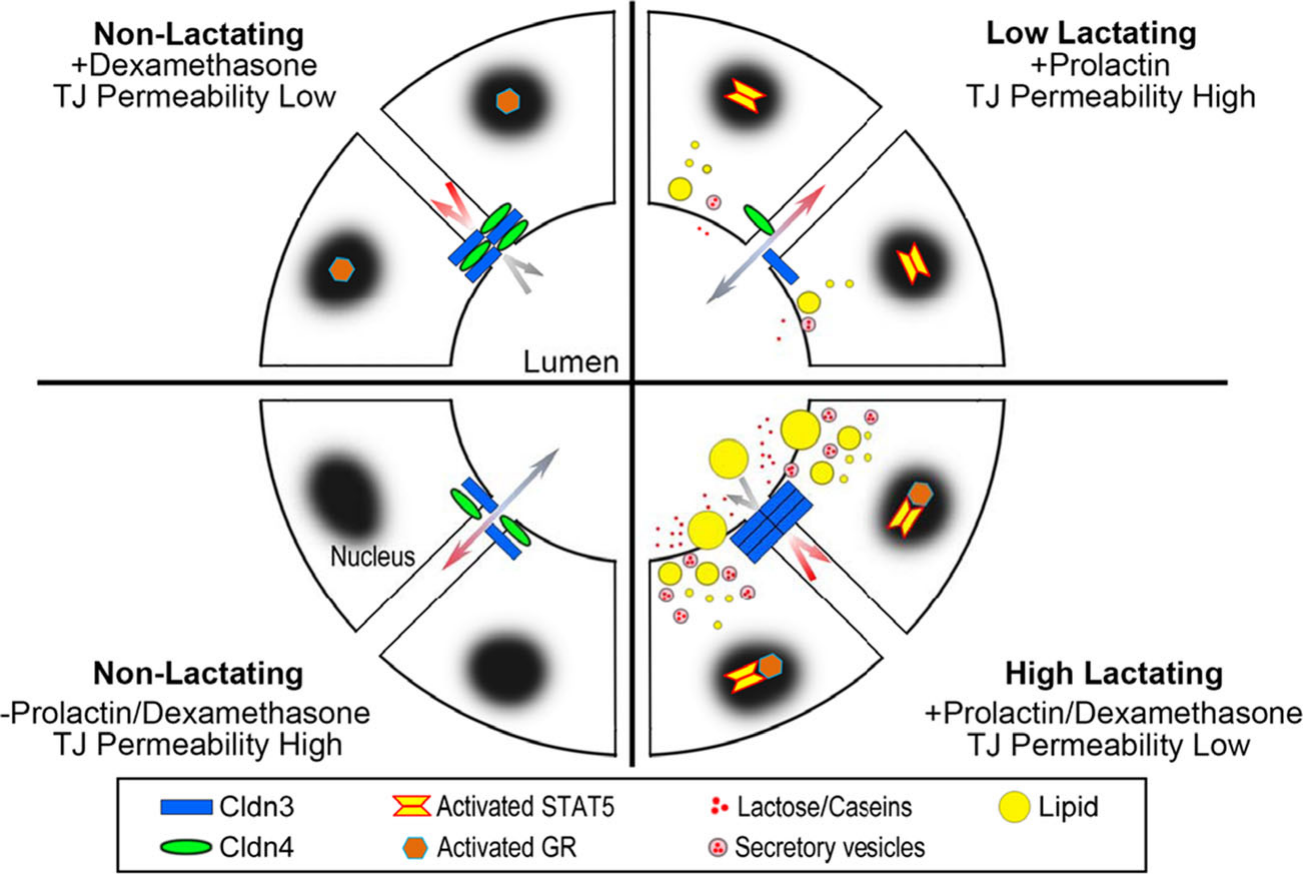
Model for hormonal regulation of claudin-3 and claudin-4 in the lactating mammary gland. Data from cultures of mouse mammary epithelial cells show that claudin-3 and claudin-4 respond to prolactin and glucocorticoids, major regulators of secretory activation in a manner similar to casein and lipid secretion [43]. See text for details

The next question was how the changes in claudin expression and localization in lactation relate to the hormonal influences that set mammary functions like casein synthesis and milk secretion in motion at parturition. Primary cells (MEC) were isolated from the mammary glands of virgin ICR mice and maintained in culture [64], In the MEC without hormones epithelial permeability was low and little claudin-3 and claudin-4 was localized to the tight junction. Addition of glucocorticoid alone promoted the barrier function of the epithelium and both claudins-3 and -4 were localized to the tight junction. When prolactin was added alone both claudins decreased and some claudin-3 appeared to move into the tight junctions. Addition of both hormones together led to increased localization of claudin-3 with occludin at the tight junctions and a marked decrease in claudin-4. β-casein was increased substantially by the addition of both hormones. The effect of glucocorticoid alone could be mimicked in the lactating gland by systemic administration of the inhibitor of prolactin secretion, bromocriptine. β-casein decreased to about 30% of the level observed at full lactation, claudin-3 and claudin-4 protein increased; both were localized to the cytoplasm. These results, modelled in Fig. 6 show definitively that claudin protein expression and localization are under the control of the same hormones that sustain milk secretion during lactation. Whether Stat5a and glucocorticoid signaling interact directly or indirectly with the regulation of the expression of the mRNA for claudins-3 and -4 is a question for future research.

### Claudins in the Involuting Mammary Gland

**Fig. 7:**
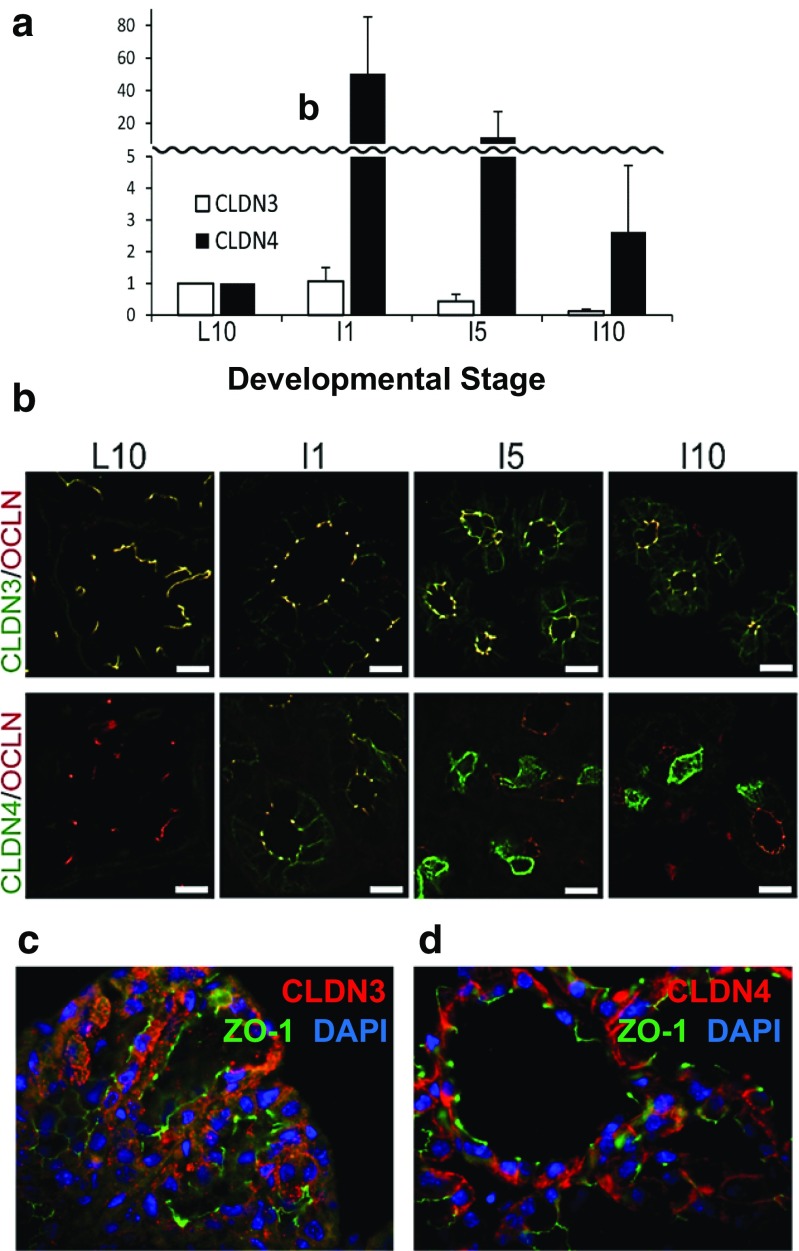
Claudins in mammary involution. **a** Levels of claudins-3 and -4 protein from Western blots during involution. Pups were removed from day 10 lactating ICR mice. Dams were sacrificed at day 10 of lactation and 1, 5 and 10 days later. Proteins from minced mammary glands were electrophoresed and visualized with appropriate antibodies (ThermoFisher) as described [43]. **b** Immunofluorescence of claudins-3 and -4 in the samples from panel **a** compared to localization of the tight junction protein, occludin (OCLN). Images from Kobayashi lab after ref. [43]. **c**,**d** Higher power images from sections of mammary glands from 10 day lactating FVB mice sacrificed 2 days after pup removal and stained using antibodies for claudin-3, claudin-4 (claudins in *red*) and the tight junction protein ZO-1 (*green*). Nuclei stained with DAPI (blue) Significant cytoplasmic stain can be observed for both claudins with little or no overlap with ZO-1. See methods for preparation of these images from the Neville laboratory

The array analyses in Fig. 2c showed a remarkable increase in the mRNA for claudins 3 and 4 during the first few days of mammary involution in the mouse, while claudin-7 mRNA remained constant. Quantitative real time PCR data from the Myal laboratory [69] showed a similar pattern for claudins-3 and -4 as does protein abundance in studies from the Kobayashi laboratory (Fig. 7a) [43]. Claudin-3 continued to localize with occludin during early involution, although it declined in abundance after days 5 and 10 (Fig. 7b). Claudin-4, not visible in the lactating gland, rose rapidly in abundance on day 1 of involution when it was localized around the basal-lateral surfaces of most cells (Fig. 7c); it continued to surround selected cells at 5 and 10 days of involution. Higher resolution images from the Neville laboratory (Fig. 7d) of the gland two days following forced involution showed significant cytoplasmic claudin-3 and claudin-4 with little overlap with the tight junction protein, ZO-1. Cumulative analyses of mRNA and protein for claudin-3 and claudin-4 from five different laboratories [29, 31, 48, 50, 69] using four different strains of mice are congruent in that these two proteins increase substantially shortly after cessation of milk removal. In addition, both proteins are largely localized to the cytoplasm in studies from both the Neville and Kobayashi laboratories (Fig. 7b-d). As we will see in the next section, similar changes were observed after an inflammatory challenge. Stein and colleagues found significant upregulation of the LPS-binding protein as well as immune related genes during involution leading them to suggest that involution represents a process akin to wound healing, a concept reinforced more recently by Schedin and colleagues [48, 70]. This concept is further validated by the data in the next section.

### Claudin Response to Intramammary Inflammation

**Fig. 8:**
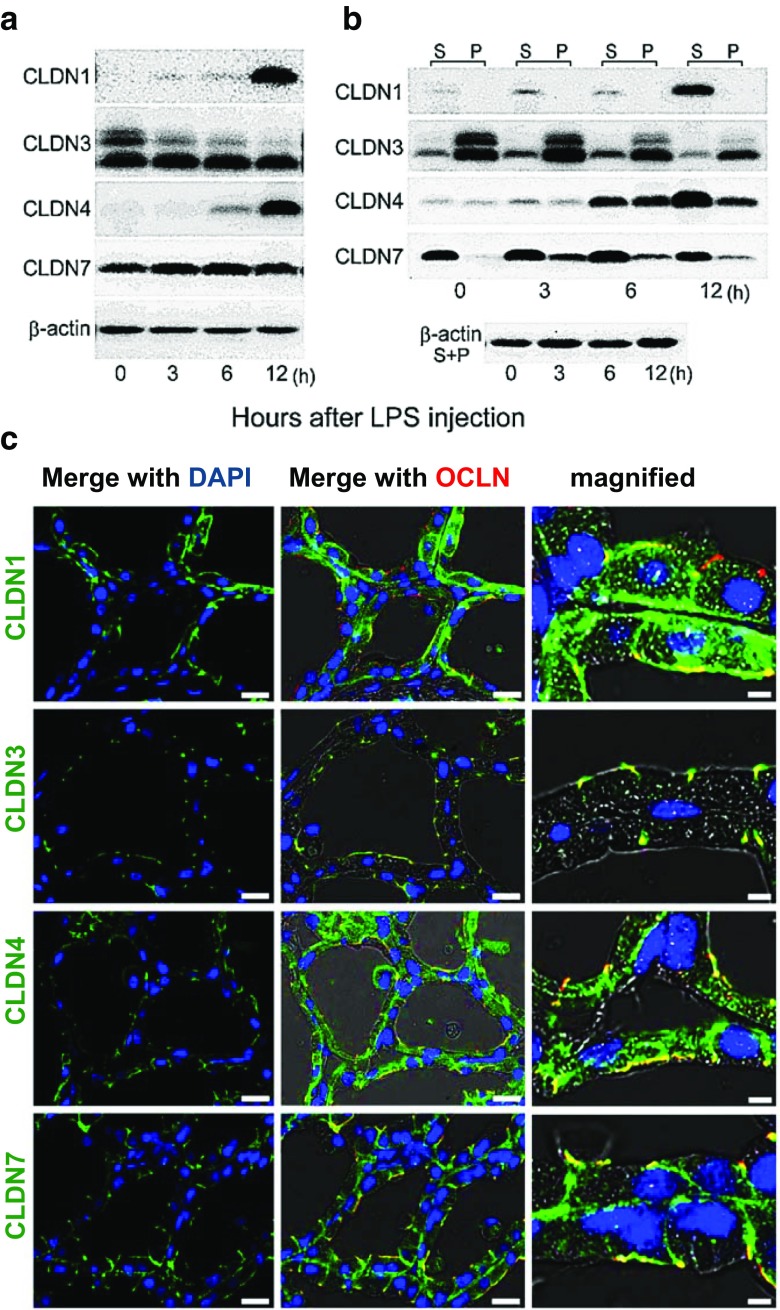
Effect of LPS on amount, type and localization of claudin protein. **a**. Western blot of claudins-1, −3, −4, and −7 in 10 day lactating gland 0, 3, 6, and 12 h after injection of *E. coli* lipopolysaccharide (LPS) into the teat canal of the fourth mammary gland of ICR mice. **b**. Western blot of claudins in Triton-X soluble (S) and insoluble (P) fractions of mammary gland lysates after LPS injection. **c**. Immunofluorescence analysis of claudins in the mammary gland 12 h after injection of LPS. Figures from ref. [31] *Plos One*

Injection of the *E. coli* endotoxin lipopolysaccharide (LPS) into the mammary gland has been used extensively as a model for mastitis, an inflammatory state of the mammary epithelium [71]. To study how inflammation affects claudin distribution and function Kobayashi and colleagues injected LPS intraductally into the 4th mammary gland of 10 day lactating ICR mice [31]. This treatment increased the permeability of the mammary epithelium to albumin within 3 h and led to an immediate decrease of high molecular weight claudin-3 (Fig. 8a). The high molecular weight form of claudin-3 was decreased 60% 3 h after LPS injection although total claudin-3 remained relatively constant. By 12 h post injection claudin-1 and claudin-4 protein were increased more than 20 fold (Fig. 8b). Claudin-1 was localized at least in part with occludin (Fig. 8c). Claudin-3 appeared to remain at the tight junction but was slightly more widely distributed than occludin. Claudin-4 was widely distributed throughout the cytoplasm and localized at least to some extent with occludin and claudin-7 relocated in part from the basal and lateral membranes to the apical region of the alveolar cells. In keeping with their redistribution to the cytoplasm [72] claudins-1 and -4 showed progressive changes in distribution between soluble and insoluble fractions over the 12 h after LPS injection. Claudin-7 remained largely in the soluble fraction after LPS treatment, in keeping with its usual location in cytoplasmic vesicles. Whether and how these changes in claudin distribution facilitate epithelial resistance to infection and repair is not known. Claudin-4 was elevated in many types of epithelial cells undergoing repair including migrating cells in the intestinal epithelium, wounded urothelial cells and salivary epithelial cells (reviewed in Ref [73]). The protein was increased in response to acute lung inflammation [74] and claudin-4 knockout mice had increased susceptibility to lung injury [75]. It appears that claudin-4 is a consistent inflammatory marker, although its function in what can be regarded as wound healing is not clear.

### Conclusions and Projections

**Fig. 9:**
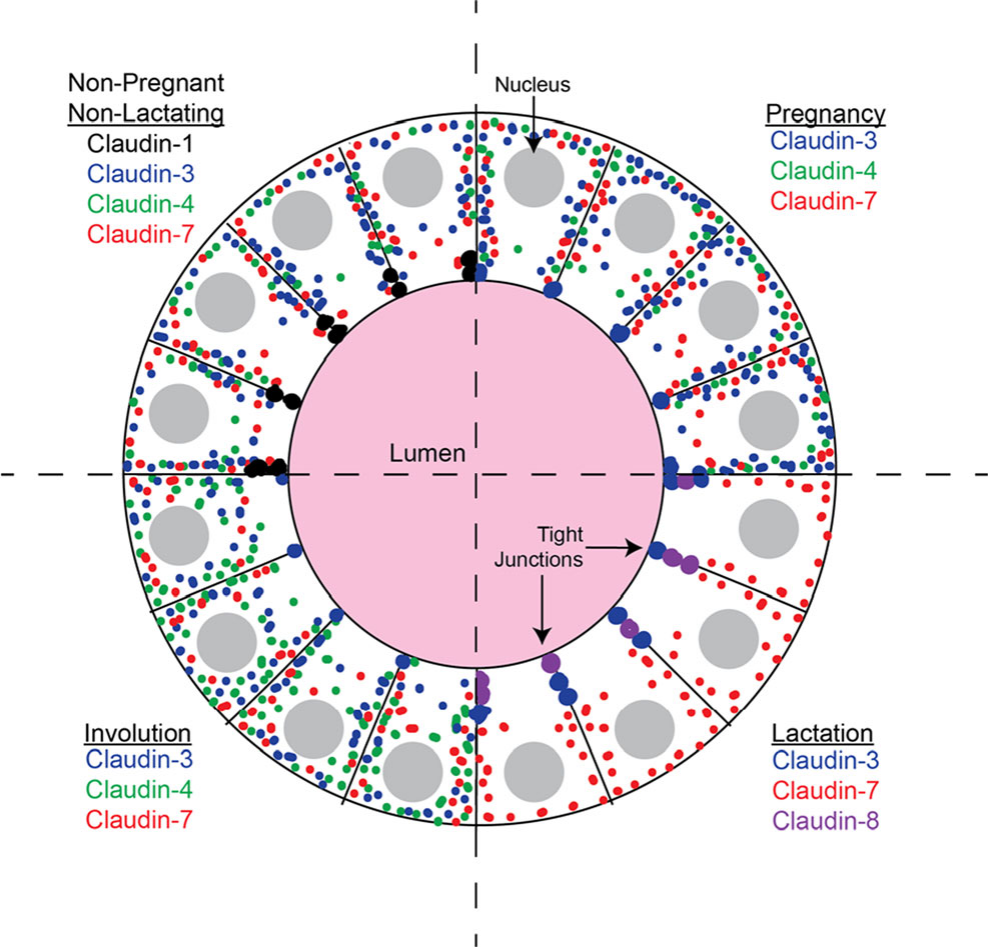
Model for distribution of claudins between cytoplasm and tight junctions at different stages of mammary development. See text for explanation

The major claudins in the mammary gland are claudins-1, -3, -4, and -7 with claudin-8 present during lactation. The distribution of these claudins between the tight junction and the cytoplasm is modelled in Fig. 9. In the non-pregnant non-lactating gland, both in virgin animals and after completion of involution, claudin-1 was the major claudin located in the tight junctions of the ductal cells. Claudins-3 and -7 were localized to numerous cytoplasmic puncta; claudin-4 was present as sparse puncta. During pregnancy claudin-3 moved to the tight junctions but was also present as a dense system of cytoplasmic puncta. Claudin-7 remained localized to the cytoplasm in puncta; sparse puncta containing claudin-4 continued to be present. During lactation both claudins-3 and -8 were localized to the tight junctions where they are presumably responsible for the high level barrier function of the epithelium. Claudin-7 continued to be present in numerous cytoplasmic puncta while claudin-4 disappeared entirely from the epithelium except for strong basolateral localization in an occasional epithelial cell. In early involution cytoplasmic claudins-3 and -4 came back robustly, presumably as part of the wound healing response of the epithelium. Claudin-8 disappeared and claudin-7 continued its cytoplasmic distribution. Claudin distribution during the inflammatory response was similar to that of early involution.

This descriptive summary of claudin distribution during mammary development provides essential information necessary for future investigations of many important questions:

1. What are the signaling mechanisms by which claudin switching is developmentally regulated? It is clear from studies of the effects of prolactin and dexamethasone on claudin distribution in isolated mammary epithelial cells [64], that claudins-3 and -4 respond to hormones in the same manner as secreted proteins like casein, but the detailed mechanisms of the hormone effects are not known.
2. What is the structure and function of the puncta containing non-junctional claudins? A start to answering this question would be to analyze the proteins with which the claudins interact, possibly using the biotin ligase technology so effectively utilized by the Anderson laboratory in MDCK cells [39].
3. Are the non-junctional claudins involved in cell-cell and cell-matrix interactions outside the tight junction? Information supporting this possibility has been reviewed in the introduction to this article.
4. What are the mechanisms by which claudins facilitate the wound healing process during involution that has been suggested in the work from the Gusterson [48] and Schedin [70] laboratories?

Importantly, the answers to these questions may also increase our understanding of the roles of claudins-3, -4 and -7 in breast cancer, where high levels of expression, not associated with tight junctions, are often observed.

## Materials and Methods for Unpublished Experiments

### Methods are Annotated by Figure Number

Figure 1. These figures were taken from the literature. The original papers should be consulted for methods.

Figure 2a. Explicit methods are given in ref [29]. Briefly female CD1 mice were sacrificed at various times during the reproductive cycle, 4th mammary glands were flash frozen after removal of the lymph node until homogenization for mRNA analysis by quantitative real time PCR for claudin-7 and murine cytokeratin 19, a marker of luminal epithelial cells.

Figure 2b, c. Stein and colleagues [48] isolated mRNA from the 4th mammary gland of 3 Balb/C mice each at 10 and 12 weeks of age, pregnancy days 1, 2, 3, 8.5, 12.5, 14.5 and 17.5, lactation days 1, 3, and 7 and involution days 1, 2, 3, 4, and 20 after forced weaning on lactation day 7. Rudolph and colleagues [49] isolated mRNA from the 4th mammary gland of 4 FVB mice each at six weeks of age, pregnancy days 1, 3, 7, 12, and 17 and lactation days 1, 2 and 9 and involution on day 2 after forced weaning. Protocols for mRNA analysis using Affymetrix Mu74Av2 microarray chips are detailed in the original publications. The data were GC-RMA normalized to correct for probeset-GC bias and raw expression values plotted (i.e. GR-RMA normalized but not Log2 transformed values, providing a rough estimate of mRNA expression amplitude). Raw data from glands isolated on similar days did not differ between mice from the two experiments so the data were combined when the time points overlapped.

Figure 3a *Mammary gland collection*: The third mammary gland on the left side of adult (>12 weeks) non-pregnant non-lactating FVB mice (Jackson Laboratories, Bar Harbor, Maine, USA) was dissected from mice euthanized by cervical dislocation after CO2 anesthesia; procedures were approved by the University of Colorado Animal Care and Use Committee.

Figure 3 b–e, Fig. 4c–f, Fig. 5d, Fig. 7d–e. *Mammary gland Collection:* CD-1 mice, purchased from Charles River Breeding Laboratory (Wilmington, DE), were maintained in the USDA approved Animal Resource Center of the University of Colorado Health Sciences Center. All procedures were approved by the Institutional Animal Care and Use Committee. The fourth mammary glands of virgin female mice at 3, 6, and 12 weeks of age, of female mice during early gestation (7 days), mid-gestation (12 days) and late gestation (18 days), at days 2 and 10 of lactation, and at day 2 involution were collected after killing with a lethal dose of pentobarbital. The day on which vaginal plugs were observed was counted as day one of pregnancy. Paraffin sections were derived as described in the previous section.

Figure 3 a–e, Fig. 4c–f, Fig. 5d, Fig. 7d–e. *Preservation of tissue and slide preparation.* Half of each tissue piece was placed in a cassette and submerged in phosphate buffered saline (PBS) supplemented with 4% paraformaldehyde overnight at 4 °C. Tissue cassettes were transported to the University of Colorado Denver Research Histology Shared Resource Facility for paraffin embedding, sectioning and deposition on microscope slides.

*Immunofluorescence.* Slides were washed twice in xylene for 20 min, twice in 100% ethanol for 3 min, and once in 90%, 70%, and 30% ethanol for 3 min each followed by washing in PBS for 5 min before antigen retrieval with Antigen Unmasking Solution (Vector Laboratories, Inc., Burlington, CA, USA). Sections were submerged in the unmasking solution and brought to a boil 10 times with a 45 min rest between each boil. After a 10 min cool-down and a 5 min wash in PBS, sections were placed in a moist chamber and permeabilized for 15 min with 1% TritonX-100 in PBS. Sections were rinsed with PBS twice before blocking for 1 h in 10% normal donkey serum in PBS supplemented with 100 μg/mL saponin. Primary antibody was then applied overnight at 4 °C. Primary antibodies included: rabbit anti-claudin-3 (1:200; Invitrogen, Carlsbad, CA, USA), rabbit anti-claudin-4 (1:200; Invitrogen), mouse anti-claudin-4 (1:200; Invitrogen), rabbit anti-claudin-7 (1:200; Invitrogen), mouse anti-claudin-7 (1:200; Invitrogen). Sections were then washed five times with PBS for 5 min before application of secondary antibody or DAPI (5 μg/ml, MP Biochemicals, Solon, OH, USA) for 45 min at room temperature. Secondary antibodies were: donkey anti-rabbit conjugated to CY3, donkey anti-mouse conjugated to FITC (all purchased from Jackson ImmunoResearch Laboratories, West Grove, PA). Sections were then washed five times with PBS before OPDA (20 mg/ml, o-phenylenediamine dihydrochloride in 1 M Tris, pH 8.5) was applied with a coverslip. Fluorescence was imaged with an Olympus Spinning Disk confocal microscope (University of Colorado AMC Light Microscopy Core).

Figure 4a, b. *Mice:* FVB mice were purchased from Jackson Laboratories, Bar Harbor, Maine, USA and maintained in the laboratory. At 8 weeks of age or older mice were bred; day 1 of pregnancy was defined as the day a postcoital plug was observed. L1 was identified as the first day litters were present. For Fig. 4a mammary glands were harvested from dams at P14 and L2. For Fig. 4b and at P13.5, P17.5, P 18.5, P19.5, during parturition and at L1, L2 and L9 . All animal procedures were approved by the Institutional Animal Care and Use Committee of the University of Colorado Anschutz Medical Campus.

Figure 4a *Mammary epithelial cell (MEC) isolation*: Adipose-depleted mouse MECs were isolated from the upper inguinal glands as described [58] with modifications. Specifically, after removal of lymph nodes, the mammary glands were removed, minced, and digested with 1 mg/mL collagenase type 1 (Worthington Biochemical Corporation, LS004196) in Dulbecco’s Modified Eagle Medium: Nutrient Mixture F-12 (HyClone, 11,330–032), for 80 min in a 37 °C rotor. Collagenase was then quenched with 0.5% fetal bovine serum, and the digested cell suspension was pelleted by centrifugation. Erythrocytes were removed by successive washes of the cell pellet in Dulbecco’s phosphate-buffered saline with calcium and magnesium (Hyclone SH30264.01) followed by 2-s centrifugations at 1500 rpm until pellet was no longer red.

*RNA isolation and hybridization*: Total RNA was isolated from MECs using Trizol solution (Thermo Fisher Scientific, Waltham, MA, USA) and purified by Qiagen miRNA columns (Qiagen, Venlo, Netherlands). RNA concentration and purity were assessed in Applied Biosytems Bioanalyzer 2100 (Thermo Fisher Scientific). A 1 μg aliquot of total RNA from each sample was labeled with FlashTag Biotin RNA Labeling kit (Genisphere, Hatfield, PA, USA) and hybridized onto GeneChip Mouse Gene 1.0 ST, GeneChip miRNA 1.0 ST (Affymetrix, Santa Clara, CA, USA) according to the manufacturer’s recommendations and performed in the University of Colorado Cancer Center Microarray Core Facility. Affymetrix CEL files from all samples were loaded on to Genespring GX10. Signal intensities for all probe sets were obtained using Robust Multichip Averaging summarization algorithm, involving three steps – background correction, quantile normalization and probe summarization (median polish). Quality control was performed by principal component analysis to identify outliers. Differentially expressed probe sets between P14 and L2 were identified by performing an unpaired t-test to obtain raw *P*-values, which were subsequently corrected by multiple testing using the BH-FDR method. A 5% FDR cut-off was chosen to identify differentially expressed probe sets. The raw data for the array used in the microarray hybridization study are available at Gene Expression Omnibus (GEO) (http://www.ncbi.nlm.nih.gov/geo) under accession number GSE87584.

Figure 4b *RNA isolation:* Fourth mammary glands were dissected from FVB mice, the lymph node removed, and the tissues snap frozen in liquid nitrogen. Frozen samples were pulverized and 100 mg was added to 1 mL of Trizol (Sigma Aldrich, St. Louis, MO) and homogenized using a Brinkman Polytron (www.brinkmann.com) on medium setting for 20–45 s. Lysates were cleared at 13,000 x G for 10 min at 4 °C and the supernatant was collected. RNA was extracted according to the manufacturer’s protocol. RNA pellets were resuspended in 40–200 μL nuclease-free water (volume dependent on pellet size), and contaminants removed using the Qiagen RNeasy mini Plus protocol according to manufacturer (kit # 74134). Total RNA was quantified using the Nanodrop 1000 spectrophotometer (Life Technologies, Grand Island, NY). Total RNA integrity was determined the Agilent 2100 Bioanalyzer Nanoscale Microfluidics Chip Assay (Wilmington, DE).

*cDNA Synthesis and Quantitative Real Time PCR:* All reagents for cDNA synthesis were purchased from Applied Biosystems (Foster City, CA). 1.0 μg of total RNA was incubated with 2.5 μL of 5.0 nmole random hexamers at 65 °C for 5 min in a total volume of 11.5 μL. Samples were placed on ice and then gently spun down. For each reaction tube, 10 μL of 25 mM MgCl_2_, 5 μL of 10X PCR Buffer A, 5 μL of 2.5 mM dNTP each, 1 μL of 20 units/μL RNAse Inhibitor, and 2.5 μL of 50 units/μL MMLV reverse transcriptase were mixed. 38.5 μL of this master mix was added to each sample and mixed by pipetting. An Applied Biosystems Gene Amp 9700 thermocycler program was: 42 °C for 60 min, 95 °C for 5 min, and 4 °C hold. Samples were placed on ice and spun down. Primer/probe sets were designed using Primer Express software (Applied Biosystems) and purchased from Integrated DNA Technologies, Coralville, IA. 5 μL of cDNA (diluted 1:4) was used as template in with 12.5 μL of 2× Universal Master Mix, 2.5 μL 20× primer/probe mix (5 μM primers, 2.5 μM probe), and 5 μL nuclease free water to make a total reaction volume of 25.0 μL. See Additional File 1 for primers and probe sets used.

Figure 4a *Statistics:* Data are presented as Mean ± Standard Error of the Mean (s.e.m.) with four samples per time point. An unpaired Student *t* test was used for statistical comparison between P14 and L2 groups. A *p* value of <0.05 was considered significant.

*Methods for the images from the Kobayashi are given in the relevant articles.*

## Electronic supplementary material

ESM 1 (DOCX 16 kb)
