# Supplementary material for: Developmental Expression of Claudins in the Mammary Gland
Source: J Mammary Gland Biol Neoplasia. 2017 Apr 28;22(2):141–57. doi: 10.1007/s10911-017-9379-6 (PMC5488167; doi:10.1007/s10911-017-9379-6)
Supplement: Supplementary file 1 — (DOCX 16 kb) [file 10911_2017_9379_MOESM1_ESM.docx]

**Additional File 1**

**Primers/Probe sets**
